# Loss of *Gata4* in Sertoli cells impairs the spermatogonial stem cell niche and causes germ cell exhaustion by attenuating chemokine signaling

**DOI:** 10.18632/oncotarget.6115

**Published:** 2015-10-14

**Authors:** Su-Ren Chen, Ji-Xin Tang, Jin-Mei Cheng, Jian Li, Cheng Jin, Xiao-Yu Li, Shou-Long Deng, Yan Zhang, Xiu-Xia Wang, Yi-Xun Liu

**Affiliations:** ^1^ State key Laboratory of Stem Cell and Reproductive Biology, Institute of Zoology, Chinese Academy of Sciences, Beijing, RP China; ^2^ University of the Chinese Academy of Sciences, Beijing, RP China

**Keywords:** Gata4, spermatogonial stem cell, Sertoli cells, niche, chemokines, Pathology Section

## Abstract

Sertoli cells, the primary somatic cell in the seminiferous epithelium, provide the spermatogonial stem cell (SSC) microenvironment (niche) through physical support and the expression of paracrine factors. However, the regulatory mechanisms within the SSC niche, which is primarily controlled by Sertoli cells, remain largely unknown. GATA4 is a Sertoli cell marker, involved in genital ridge initiation, sex determination and differentiation during the embryonic stage. Here, we showed that neonatal mice with a targeted disruption of *Gata4* in Sertoli cells (*Gata4*^flox/flox^; *Amh*-Cre; hereafter termed *Gata4* cKO) displayed a loss of the establishment and maintenance of the SSC pool and apoptosis of both gonocyte-derived differentiating spermatogonia and meiotic spermatocytes. Thus, progressive germ cell depletion and a Sertoli-cell-only syndrome were observed as early as the first wave of murine spermatogenesis. Transplantation of germ cells from postnatal day 5 (P5) *Gata4* cKO mice into *Kit*^W/W-v^ recipient seminiferous tubules restored spermatogenesis. In addition, microarray analyses of P5 *Gata4* cKO mouse testes showed alterations in chemokine signaling factors, including *Cxcl12*, *Ccl3*, *Cxcr4* (CXCL12 receptor), *Ccr1* (CCL3 receptor), *Ccl9*, *Xcl1* and *Ccrl2*. Deletion of *Gata4* in Sertoli cells markedly attenuated Sertoli cell chemotaxis, which guides SSCs or prospermatogonia to the stem cell niche. Finally, we showed that GATA4 transcriptionally regulated *Cxcl12* and *Ccl9*, and the addition of CXCL12 and CCL9 to an in vitro testis tissue culture system increased the number of PLZF^+^ undifferentiated spermatogonia within *Gata4* cKO testes. Together, these results reveal a novel role for GATA4 in controlling the SSC niche via the transcriptional regulation of chemokine signaling shortly after birth.

## INTRODUCTION

Approximately 50% of human infertility is attributable to male defects, of which 70-90% stem from impaired spermatogenesis [1]. The process of spermatogenesis can be divided into three phases: mitotic proliferation of spermatogonia, meiotic division of spermatocytes and morphologic differentiation of haploid spermatids during spermiogenesis. Mouse spermatogenesis is initiated only a few days after birth and proceeds in a synchronized manner [2]. Key time points regarding the appearance of particular germ cell types are well defined during the first cycle of spermatogenesis [3, 4]. Postnatal day (P) 5-7 mouse testes contain only Sertoli cells and gonocyte-derived spermatogonia in the seminiferous tubules. Early spermatocytes appear at P9, the pachytene stage of the first meiotic prophase is initiated at P14, round spermatids are generated at P20, and condensing spermatids are created by P30 [3, 5].

Spermatogenesis requires the proper regulation of spermatogonial stem cells (SSCs) to replenish the testis with germ cell progenitors during adult life. Sertoli cells, the primary somatic cell type in the seminiferous epithelium, directly interact with SSCs to control their proliferation and differentiation through the secretion of specific factors (reviewed in [6]). Glial cell line-derived neurotrophic factor (GDNF), which is secreted by Sertoli cells, is a well-defined paracrine factor that promotes SSC self-renewal and maintenance in the niche [7]. Supplementation with fibroblast growth factor 2 (FGF2, also known as basic FGF (bFGF)), which is also secreted by Sertoli cells, in combination with GDNF allows for the long-term self-renewal and expansion of SSCs [8]. CXCL12 encodes a chemokine that is expressed and secreted by Sertoli cells and binds to the CXCR4 receptor on SSCs to regulate their self-renewal and maintenance [9]. Chen et al. demonstrated that Ets-related molecule (ERM; also known as ETV5) is primarily expressed by Sertoli cells and is required for SSC self-renewal because it regulates several chemokine genes, including CXCL12, CXCL5, and CCL7 [10, 11]. However, apart from these involved genes, the mechanisms by which Sertoli cells regulate the SSC niche are largely unknown.

The GATA transcription factor family consists of six members (GATA1-6). The members share the highest homology in the zinc finger DNA-binding domain, and they all bind to the consensus site (T/A)GATA(A/G). Many Sertoli cell-specific genes have GATA-binding sites in their promoters [12]. Lindeboom et al. suggested that GATA1 in Sertoli cells is not essential for murine testis development or spermatogenesis [13].

GATA4 is an evolutionarily conserved zinc finger transcription factor that is essential for the early development of multiple organs, including the heart, foregut, liver, and pancreas [14-16]. GATA4 is predominantly expressed in somatic cells within the testis (Sertoli cells, Leydig cells and other interstitial cells), and its expression remains consistently abundant in both embryonic gonads and adult testes. Recently, Hu et al. suggested that mouse embryos that were conditionally deficient for *Gata4* showed no signs of gonadal initiation [17]. In XY transgenic mice harboring mutant GATA4 (*Gata4^ki^*) that abrogates GATA4 binding to the co-factor FOG2 (Friend of GATA), genital ridges formed, but the subsequent differentiation into testes was blocked, and SRY (Sex Determining Region, Y Chromosome) expression was attenuated [18]. Mouse embryos that were heterozygous for *Gata4^ki^* in specific genetic backgrounds also showed sex reversal from genetic males to phenotypic females [19]. An *in vitro* study further suggested that GATA4 and WT1 (Wilms’ Tumor 1) synergistically activate the transcription of *Sry* [20]. Manuylov et al. suggested that GATA4 regulates testicular differentiation. The excision of *Gata4* by *Wt1^CreERT2^* at E10.5 led to an early and broad failure of Sertoli cell differentiation and male development with concurrent sex reversal. Furthermore, *Sf1*-Cre-mediated excision of *Gata4* at E12.5 led to testis cord defects and a loss of *Dmrt1* gene expression in Sertoli cells [21].

The critical role of GATA4 in human gonadal development is highlighted by a familial case of 46, XY DSD (Disorder of Sex Development) associated with a heterozygous *GATA4* p.Gly221Arg mutation [22]. The p.Gly221Arg mutant protein fails to bind to FOG2 and disrupts the synergistic activation of the *AMH* promoter. Recently, Bashamboo et al. identified three missense mutations (p.S402R, p.R260Q and p.M544I) in *FOG2*, which abolished the interaction with GATA4, in two patients with 46, XY DSD [23].

Collectively, previous studies suggest that GATA4 is essential for proper fetal testis development. Here, we extended these findings by determining the effects of GATA4 expression in Sertoli cells in the postnatal testes. This study was accomplished by crossing *Amh*-Cre knock-in mice with *Gata4*^flox/flox^ mice. *Gata4* cKO males exhibited few GFRA1^+^ and PLZF^+^ (also known as ZBTB16) undifferentiated spermatogonia (including SSCs) after birth. Markers of differentiating spermatogonia (c-KIT) and meiotic spermatocytes (STRA8) exhibited normal expression, indicating ‘normal’ spermatogenic differentiation of gonocyte-derived differentiating spermatogonia in *Gata4* cKO testes; however, these cells ultimately underwent apoptosis. During the first wave of spermatogenesis, the mutant testes exhibited an extensive loss of germ cells, including SSCs, followed by a Sertoli-cell-only syndrome. Interestingly, the transcriptional levels of many chemokine signaling molecules were significantly reduced in the *Gata4* cKO testes. Furthermore, we showed that GATA4 transcriptionally regulated *Cxcl12* and *Ccl9* in Sertoli cells. The addition of CXCL12 and CCL9 to an *in vitro* testis tissue culture system significantly increased the number of PLZF^+^ undifferentiated spermatogonia in *Gata4* cKO males. Collectively, we conclude that GATA4 in Sertoli cells governs the establishment and maintenance of a SSC niche by regulating chemokine signaling.

## RESULTS

### Sertoli cell-specific knockout of *Gata4* results in a complete loss of germ cells

**Figure 1:**
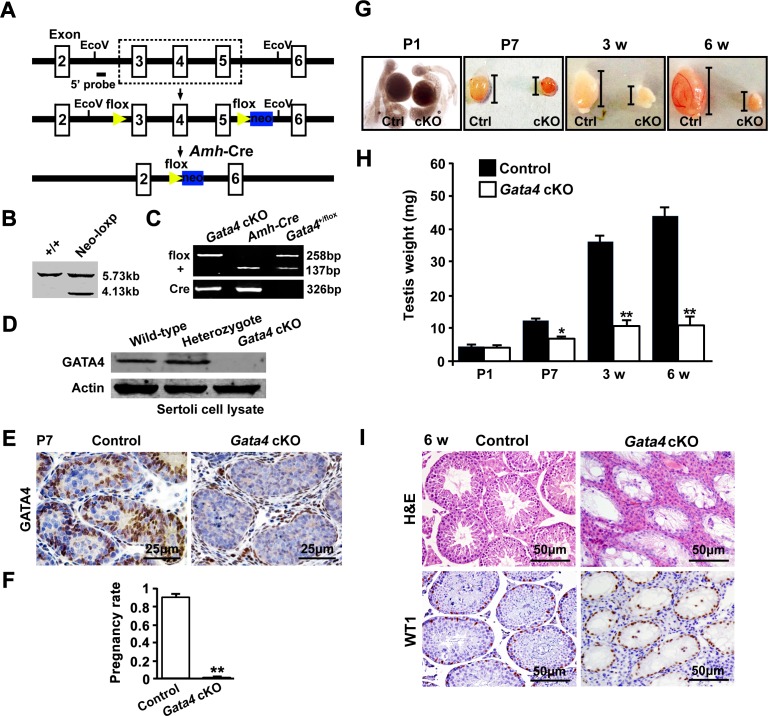
Conditional deletion of *Gata4* in Sertoli cells using Amh-Cre **A.** Structures of the floxed (*Gata4*^flox^) and null (*Gata4* cKO) *Gata4* alleles after *Amh*-Cre-mediated recombination. Yellow triangles represent loxP cassettes, and blue rectangles depict neo sites. The exons are numbered. **B.** Southern blot: EcoRV-digested ES cell DNA probed with a 5′ probe yielded a 5.73 kb fragment in recombinant animals in addition to the wild-type 4.13 kb fragment, confirming the insertion of the targeting construct into the *Gata4* locus. **C.** Genotyping via PCR. PCR analysis of the presence of the floxed and wild-type *Gata4* alleles. An analysis of the presence of Cre in the *Amh*-Cre transgene is shown below. The product size is listed. **D.** Western blot analysis of GATA4 in isolated Sertoli cell extracts from neonatal wild-type, heterozygous and *Gata4* cKO mice. Actin served as a protein loading control. **E.** Immunohistochemical staining of GATA4 in wild-type and *Gata4* cKO testes. Note that the GATA4 signal (brown) was specifically deleted in the Sertoli cells of *Gata4* cKO testes. Scale bar, 25 μm. **F.** Fertility test: 95.01 ± 1.46% of the plugged females were pregnant after crossing with *Gata4*^flox/flox^ control males, whereas none of the plugged females were pregnant after crossing with *Gata4* cKO males. The data are expressed as the mean±S.D. (*n* = 10 per genotype). **p < 0.01. **G.** Gross morphology in control (left) and *Gata4* cKO (right) testes at P1, P7, 3 w and 6 w. **H.** Histograms of testis weight in control and *Gata4* cKO mice (mg). The data are expressed as the mean±S.D. (*n* = 6 per genotype). *p < 0.05, **p < 0.01. **I**. H&E staining and immunohistochemistry of WT1 (a Sertoli cell marker; brown signals) in testes from control and *Gata4* cKO mice at 6 w. Scale bar, 50 μm.

To investigate the role of GATA4 expression in Sertoli cells during postnatal testicular development and spermatogenesis, we generated a Sertoli cell-specific *Gata4* knockout mouse line (*Gata4*^flox/flox^, *Amh*-Cre, hereafter referred to as *Gata4* cKO) by crossing a Sertoli cell-specific Cre line (*Amh*-Cre) with a *Gata4*-loxP line (Figure 1A-1C). In neonatal *Gata4* cKO mice, GATA4 was specifically inactivated in Sertoli cells, as evidenced by Western blot (Figure 1D) and immunohistochemistry (Figure 1E). The fertility of the male mice was assessed by mating 6- to 8-week-old male *Gata4* cKO and their control littermates with wild-type (C57BL/6) females over a 3-month period. As shown in Figure 1F, the *Gata4* cKO male mice were completely infertile. An examination of juvenile and adult male testes revealed no difference in fresh tissue size at postnatal day 1 (P1); however, the testes from *Gata4* cKO males at P7 or older were significantly smaller, such that by adulthood (6 weeks of age), the *Gata4* cKO testes had dramatically shrunk (Figure 1G). The testis weight of *Gata4* cKO males was significantly lower than that of wild-type males at P7, 3 weeks and 6 weeks (Figure 1H). Histological examination of 6-week-old *Gata4* cKO testes revealed that all of the tubules were devoid of germ cells and contained only morphologically normal Sertoli cells (Figure 1I).

### Sertoli-cell-only phenotype occurs as early as the initial wave of spermatogenesis

**Figure 2:**
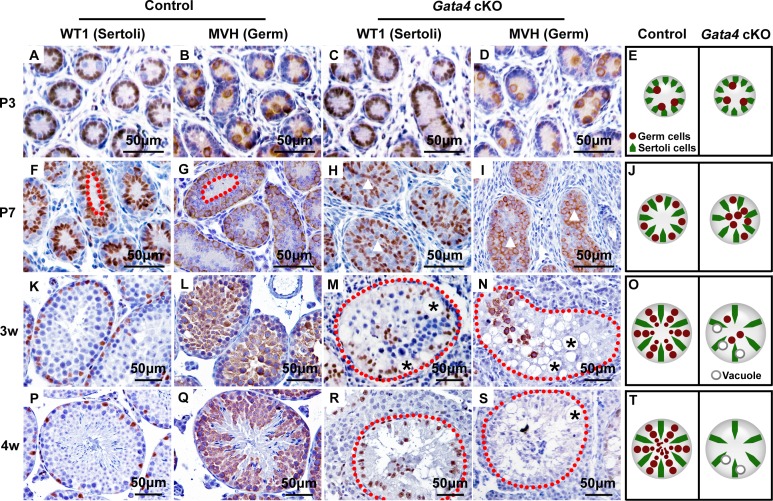
Morphological change at P7 and Sertoli-cell-only syndrome at 4 w in *Gata4* cKO testes **A.** Representative seminiferous tubule morphology at P3 **A.**-**D.**, P7 **F.**-**I.**, 3 w **K.**-**N.** and 4 w **P.**-**S.** with immunohistochemical staining of WT1 (a Sertoli cell marker, brown) and MVH (a germ cell marker, brown). The nuclei are stained blue with hematoxylin. The red dotted lines in **F.** and **G.** indicate the lumen formed by the localization of germ cells to the basement membrane in control testes. The white triangles in **H.** and **I.** indicate that no lumen formed in *Gata4* cKO testes. The red dotted lines in **M.**, **N.**, **R.** and S indicate the borders of the testicular tubules. The black stars in **M.**, **N.** and S indicate vacuoles. **E.**, **J.**, **Q.** and **T.** are cartoons that depict the differing morphologies of testicular tubules in control and *Gata4* cKO testes. Scale bar, 50 μm.

To track the key moments of spermatogenic failure, we investigated testicular morphology at four time points during the first wave of murine spermatogenesis. At P3, the seminiferous tubules of control and *Gata4* cKO mice appeared similar, with WT1-positive Sertoli cells and MVH-positive germ cells indicating the normal initiation of spermatogenesis (Figure 2A-2E). At P7, the germ cells within control testes had migrated to the basement membrane. In contrast, the germ cells of *Gata4* cKO testes remained aligned at the luminal side of the testicular tubules, indicating that they had lost the ability to locate the niche (Figure 2F-2J). Subsequently, we observed a phenotype corresponding to an age-dependent loss of germ cells in *Gata4* cKO testes. By 3 weeks, *Gata4* cKO tubules exhibited vacuolization, and no germ cells remained attached to the basement membrane (Figure 2K-2O), indicating exhaustion of the SSC pool. Consequently, *Gata4* cKO testes were entirely devoid of germ cells by 4 weeks, forming a Sertoli-cell-only phenotype (Figure 2P-2T). Accordingly, we concluded that spermatogenesis was completely disrupted as early as the first wave in *Gata4* cKO testes. We speculate that GATA4 plays a critical role in the establishment and/or maintenance of the spermatogonial progenitor pool.

### Failure to establish and maintain the SSC pool in *Gata4* cKO testes

**Figure 3:**
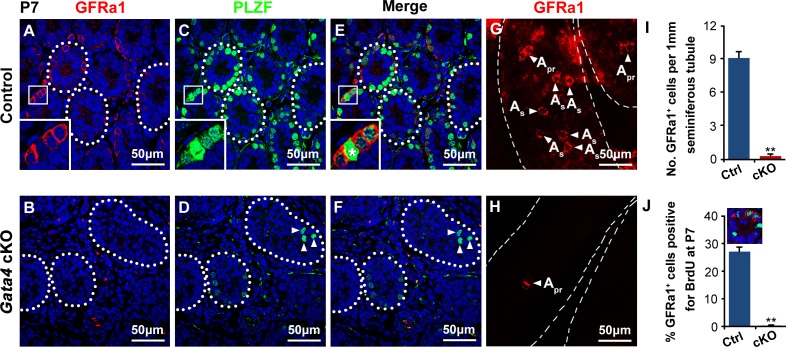
Few GFRa1^+^ or PLZF^+^ spermatogonial progenitors within the *Gata4* cKO testes **A., B.** Testis sections from control and *Gata4* cKO mice at P7 were probed with antisera against GFRa1 (red). The nuclei are counterstained with DAPI (blue). **C., D.** Immunofluorescence staining for PLZF (green) in control and *Gata4* cKO testis sections at P7. The white arrowheads in **D.** indicate the low number of PLZF^+^ spermatogonia within *Gata4* cKO testes. The white dotted lines indicate the testicular tubules. Scale bar, 50 μm. **E.** and **F.** are the merged images of A+B and C+D, respectively. **G.**, **H.** Representative images of staining for GFRa1 (red)-expressing cells in a whole-mount seminiferous tubule at P7. The white arrowheads indicate A_single_ and A_paired_ spermatogonia according to their morphology. **I.** Density of GFRa1^+^ spermatogonia per 1 mm in seminiferous tubules of the control and *Gata4* cKO testes (*n* = 3 each). The data are expressed as the mean±S.D. **p < 0.01. **J.** BrdU-labeling index of GFRa1^+^ spermatogonia in control and *Gata4* cKO testes at P7. BrdU was injected 4 h before the mice were sacrificed. The data are expressed as the mean±S.D. **p < 0.01.

To further explore the premature exhaustion of the spermatogonia progenitor pool in *Gata4* cKO testes, we measured the abundance of undifferentiated spermatogonia at P7 by staining histological sections and whole-mount tubules for PLZF and GFRa1. PLZF is expressed at all stages in undifferentiated spermatogonia, whereas GFRa1 is expressed primarily in A_single_ (so-called SSCs) and A_paired_ spermatogonia [24-26]. At P7, the density of GFRa1^+^ spermatogonia was significantly decreased in *Gata4* cKO testes compared to control testes (Figure 3A, 3B, 3G-3I). Similarly, PLZF^+^ spermatogonia were rare in *Gata4* cKO tubules but abundant in age-matched controls (Figure 3C, 3D). Upon mitotic division, SSCs can self-renew to maintain the spermatogonia progenitor pool. Next, a BrdU incorporation assay was conducted to test the self-renewal characteristics of the extremely insufficient population of GFRa1^+^ spermatogonia in P7 *Gata4* cKO testes. We found that approximately 28% of the GFRa1^+^ spermatogonia incorporated BrdU in control testes, whereas no GFRa1^+^ /BrdU^+^ spermatogonia were observed in *Gata4* cKO testes (Figure 3J). Collectively, these data suggest that neither the establishment nor the maintenance of the SSC pool is appropriately regulated in *Gata4* cKO testes.

### Fate of gonocyte-derived differentiating spermatogonia during the first wave of spermatogenesis in *Gata4* cKO testes

**Figure 4:**
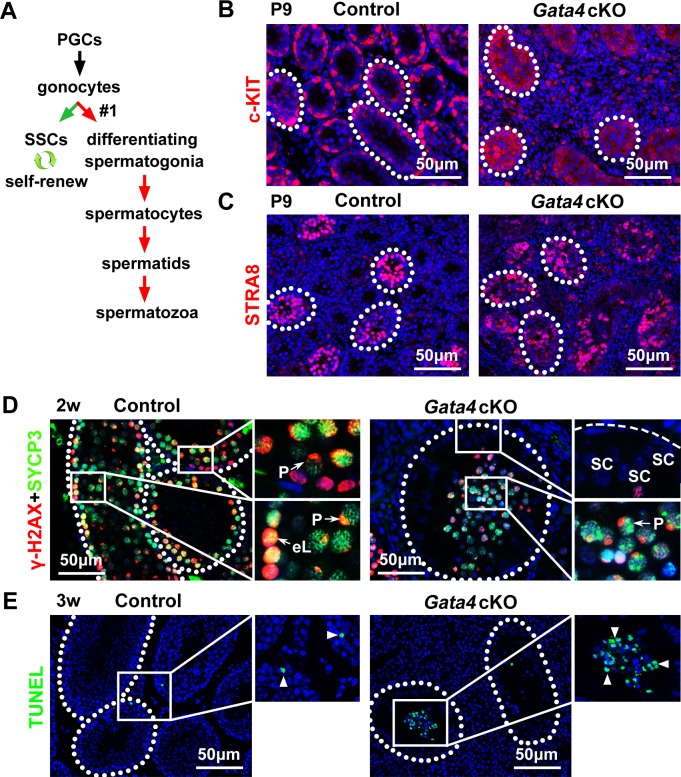
Differentiation of gonocyte-derived differentiating spermatogonia but subsequent apoptosis of meiotic spermatocytes in *Gata4* cKO testes **A.** Model for the two lineages in murine spermatogenesis: gonocyte-derived undifferentiated spermatogonia (also named SSCs) and gonocyte-derived differentiating spermatogonia. The distribution of c-KIT **B.** and STRA8 **C.** in testes from control and *Gata4* cKO mice at P9. **D.** γ-H2AX (red) and SYCP3 (green) immunostaining of control and *Gata4* cKO testes at 2 w. eL, early leptotene; P, pachytene; SC, Sertoli cells. **E.** A TUNEL assay was performed to detect apoptotic cells (green; arrowheads) in control and *Gata4* cKO testes at 3 w. The white dotted lines in **B.**-**E.** indicate the testicular tubules. *Scale bars* in **B.**-**E.**, 50 μm.

During the first wave of spermatogenesis, gonocytes can directly give rise to differentiating spermatogonia (unique in the first wave of spermatogenesis) and SSCs in parallel [2] (Figure 4A). As SSCs exhibited defects in both establishment and maintenance in *Gata4* cKO testes (Figure 3), we examined the fate of the gonocyte-derived differentiating spermatogonia in *Gata4* cKO testes. c-KIT staining of differentiated spermatogonia (Figure 4B) and STRA8 staining of meiotic spermatocytes (Figure 4C) were similar in control and *Gata4* cKO testes at P9, suggesting that the differentiation of gonocyte-derived differentiating spermatogonia was not disrupted by GATA4 deletion in Sertoli cells. Furthermore, double immunofluorescence staining of meiosis-associated markers, such as γ-H2AX and SYCP3, was performed at 2 weeks. As shown in Figure 4D, spermatocytes in the pachytene stage were observed in both control and *Gata4* cKO tubules. In addition, Western blotting showed that the protein levels of PLZF and GFRa1 were significantly reduced, while the expression of c-KIT, STRA8 and SYCP3 was not altered in *Gata4* cKO testes compared to control testes (Figure S1). To determine whether massive spermatocyte cell death led to the exhaustion of germ cells in *Gata4* cKO testes, we assayed apoptosis via a TUNEL assay at 3 weeks. As expected, massive germ cell apoptosis was observed in *Gata4* cKO tubules (Figure 4E).

### Transplantation of germinal cells from *Gata4* cKO donors into *Kit*^W/W-v^ recipients

**Figure 5:**
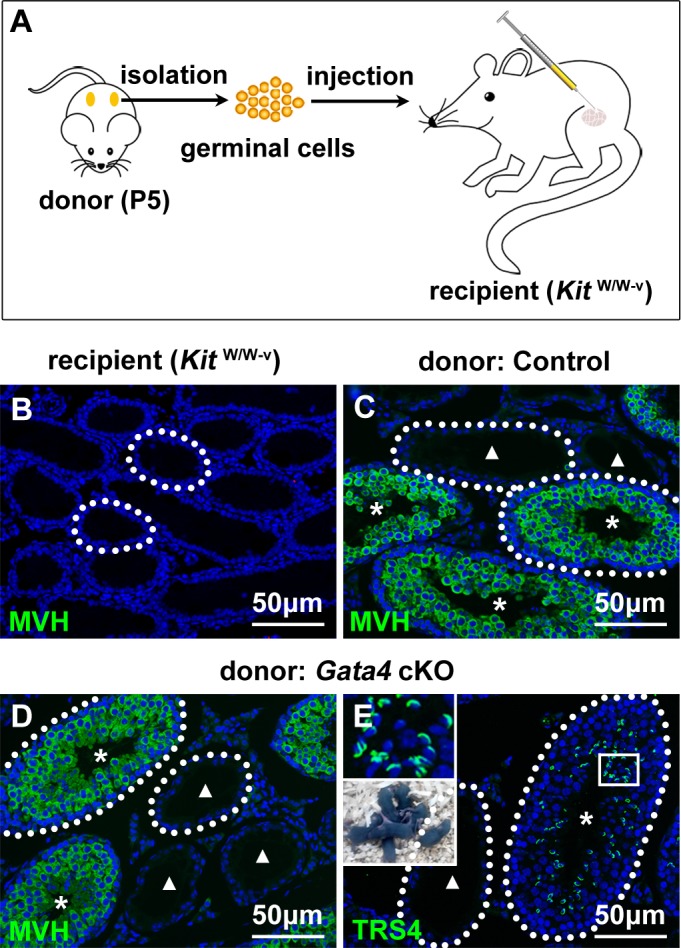
Histological images of seminiferous tubules in recipient mouse testes after transplantation of germinal cells from P5 *Gata4* cKO males **A.** Schematic of the germinal cell transplantation strategy. **B.** Histological section of *Kit*^W/W-v^ recipient seminiferous tubules stained with an anti-MVH antibody (green). Representative images of MVH immunostaining (green) of *Kit*^W/W-v^ recipient seminiferous tubules after transplantation with donor germinal cells from control **C.** and *Gata4* cKO males **D.** Stars and triangles indicate colonized and noncolonized seminiferous tubules after transplantation, respectively. **E.** A testicular cross section of *Kit*^W/W-v^ recipient testes at 8 w after transplantation of donor germinal cells from *Gata4* cKO males stained with an anti-TRS4 antibody (green) to show the acrosome. The transplanted *Kit*^W/W-v^ recipient males reproduced spermatozoa with acrosome (above) and generated progeny (below). The cell nuclei are stained with DAPI (blue). The white dotted lines indicate the testicular tubules. *Scale bars* in B-E, 50 μm.

Because *GATA4* mutation leads to male sterility [22], we attempted to rescue the infertile phenotype of the *Gata4* cKO mice and thereby identify potential new leads for the treatment of infertile patients carrying a *GATA4* mutation. We transplanted germinal cells from 5-day-old control and *Gata4* cKO testes into the seminiferous tubules of *Kit*^W/W-v^ mice (Figure 5A). The testes of *Kit*^W/W-v^ mice are hospitable to donor cell colonization because they lack endogenous germ cells and have functionally normal Sertoli cells (Figure 5B). We showed that the transplanted germinal cells from control (Figure 5C) and *Gata4* cKO (Figure 5D) males differentiated into MVH-positive spermatocytes and round spermatids in the seminiferous tubules of *Kit*^W/W-v^ recipient mice after transplantation. In addition, TRS4-positive round and elongating spermatids were aligned at the luminal side of the seminiferous tubules in *Kit*^W/W-v^ recipient testes by 8 weeks after transplantation. Offspring were produced from germ cells from transplanted *Kit*^W/W-v^ recipient males via *in vitro* microinsemination (Figure 5E). These results suggested that GATA4 functions in Sertoli cells to establish the proper microenvironment for spermatogenesis and that transplantation provides an approach for restoring fertility in *Gata4* cKO males.

### Deletion of *Gata4* in Sertoli cells markedly attenuates Sertoli cell chemotaxis

**Figure 6:**
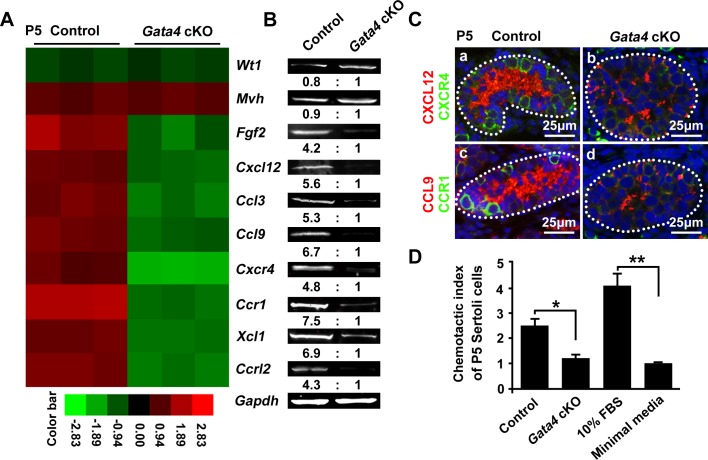
Identification of altered chemokine-related genes in *Gata4* cKO testes at P5 **A.** A heat map analysis of control and *Gata4* cKO (*n* = 3 each) testes at P5. Geen and red represent low and high expression levels, respectively. **B.** RT-PCR analyses of the indicated genes in control and *Gata4* cKO testes (*n* = 3 each) at P5. The numbers at the bottom represent the relative fold reduction measured via the density analysis. *Wt1* is a marker of Sertoli cells, and *Mvh* is a marker of germ cells. *Gapdh* served as the internal control gene. **C.** Representative images of immunofluorescent staining for CXCL12 (red) and CXCR4 (green) expression in cross-sections of testes from control (a) and *Gata4* cKO (b) mice at P5. Expression of CCL9 (red) and CCR1 (green) within the tubules of control (c) and *Gata4* cKO (d) testes at P5. DAPI was used to stain DNA. The white dotted lines indicate the testicular tubules. Scale bar: 25 μm. **D.** The ability of stem/progenitor spermatogonia to migrate toward Sertoli cells was expressed as the chemotactic index. The chemotactic index with respect to the 10% FBS positive control was approximately three-fold greater compared to the negative control. The chemotactic index of the *Gata4*-deficient Sertoli cells was not significantly different than that of the negative control, which corresponded to only minimal media. The data are expressed as the mean±S.D. *p < 0.05, **p < 0.01.

To explore the reasons for the impaired SSC niche in *Gata4* cKO testes, we conducted a microarray analysis to compare gene expression between control and *Gata4* cKO testes at P5, when the testes of the two phenotypes have an identical histological appearance. These analyses revealed significant (at least 2-fold) increases in the abundance of 64 transcripts and decreases in the abundance of 68 transcripts in *Gata4* cKO testes compared to control testes (Table S3, S4). Based on pathway term analysis, the most significant functions associated with the differentially expressed genes in *Gata4* cKO testes were chemotaxis, cell apoptosis, Sertoli cell transcription factor, cell adhesion and extracellular matrix, fatty acid metabolism and RA metabolism. Notably, the expression of many genes involved in chemokine signaling, including *Cxcl12*, *Ccl3*, *Ccl9*, *Cxcr4*, *Ccr1*, *Xcl1* and *Ccrl2*, was significantly down-regulated following *Gata4* gene deletion (Figure 6A). *Cxcl12*, *Ccl3*, *Ccl9*, *Xcl1* and *Ccrl2* were expressed in Sertoli cells, while *Cxcr4* and *Ccr1* were present in germ cells (Figure S2). The microarray results were confirmed by RT-PCR analysis of testes from P5 control and *Gata4* cKO littermates (Figure 6B). To further evaluate the expression of CXCL12, CXCR4 (CXCL12 receptor), CCL9 and CCR1 (CCL9 receptor) in the developing testis, we conducted double immunofluorescence staining of P5 mouse testis sections using specific antibodies. In wild-type testes, CXCL12 and CCL9 staining was observed within the cytoplasm of Sertoli cells, and their receptors CXCR4 and CCR1 were localized at the surface of spermatogonia (Figure 6C-a, c). In contrast, the expression of these chemokines and their receptors was markedly reduced in age-matched *Gata4* cKO testes (Figure 6C-b, d).

Because CXCL12 and CCL9 facilitate Sertoli cell chemoattraction of spermatogonia by binding to CXCR4 and CCR1 on the surface of spermatogonia [6, 9], we speculated that Sertoli cells in *Gata4* cKO testes would have decreased chemotactic activity. Thus, we analyzed the chemotactic index, which was defined as the number of spermatogonia (or other germ cells) that had migrated toward Sertoli cells. This index was significantly decreased for the migration of germ cells from P5 males toward *Gata4*-deficient Sertoli cells compared to control Sertoli cells (Figure 6D). This result indicated that *Gata4* deletion in Sertoli cells markedly attenuated the ability of Sertoli cells to chemotactically guide SSCs or prospermatogonia to the stem cell niche.

### Regulation of CXCL12 and CCL9 by GATA4 in Sertoli cells

**Figure 7:**
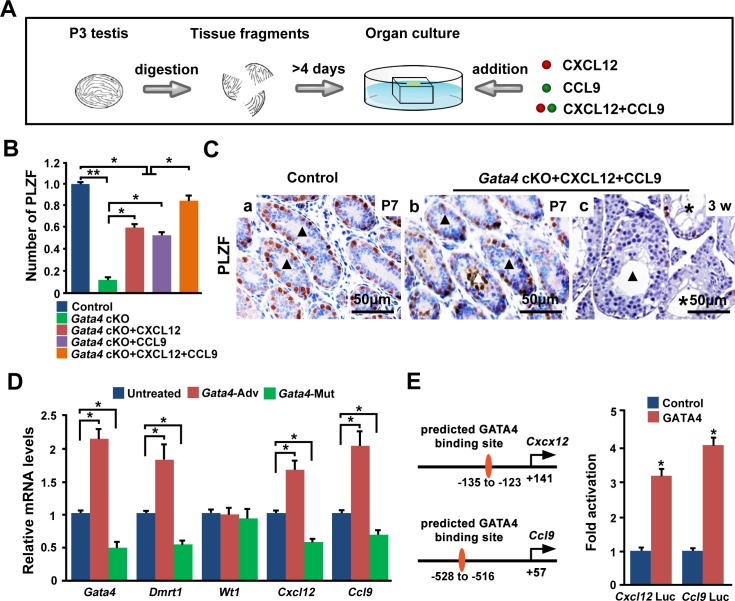
*GATA4* regulates *Cxcl12* and *Ccl9* in Sertoli cells **A.** Schematic of the *in vitro* testis tissue culture system and the chemokine rescue strategy. The tissue fragments of P3 testes were placed on an agarose gel that was half-soaked in the medium and cultured 4 or more days. CXCL12 and/or CCL9 (final concentration: 100 ng/ml) were added to the culture medium. **B.** The relative number of PLZF^+^ undifferentiated spermatogonia. Tissue fragments of P3 testes were cultured *in vitro* for 4 days, and PLZF^+^ cells were counted in three random 250 μm×250 μm areas. The data are expressed as the mean±S.D (*n* = 3 per group). *p < 0.05. **C.** A representative image of seminiferous tubule staining for PLZF (brown) after culture for 4 days (P7) (a: control; b: *Gata4* cKO) or 4 weeks (c: *Gata4* cKO). Black triangles in a and b indicate normal testicular tubules. The white triangle in b indicates a testicular tubule, where PLZF^+^ spermatogonia remain aligned along the luminal side. The black triangle in c shows tubules with germ cells arrested as spermatocytes. The white stars in c indicate tubules devoid of germ cells. Scale bar, 50 μm. **D.** The relative mRNA levels of *Gata4*, *Dmrt1*, *Wt1*, *Cxcl12* and *Ccl9* in untreated Sertoli cells, Sertoli cells transfected with a *Gata4*-expressing adenovirus, and *Gata4*^Cys294Ala^ plasmid-transfected Sertoli cells from P5 testes. The data are expressed as the mean±S.D. *p < 0.05. **E.** TM4 cells were cotransfected with *Cxcl12* Luc or *Ccl9* Luc and PCB6+GATA4 or the control PCB6+ vector. The *Cxcl12* and *Ccl9* promoters were both transactivated by GATA4. The data are present as the mean±SEM. *p < 0.05.

To determine whether the attenuated expression of CXCL12 and CCL9 caused the defect in the SSC niche in *Gata4* cKO testes, P3 testis explants from *Gata4* cKO males were cultured *in vitro*, and chemokines (CXCL12 and/or CCL9) were added to the culture medium (Figure 7A). After a 4-day culture, the number of PLZF^+^ undifferentiated spermatogonia was significantly higher after the addition of CXCL12, CCL9, or both. Moreover, the number of PLZF^+^ undifferentiated spermatogonia was significantly higher after treatment with CXCL12 and CCL9 than after treatment with CXCL12 or CCL9 individually (Figure 7B). Although some spermatogonia remained aligned at the luminal side in some testicular tubules (white triangle), the number of undifferentiated spermatogonia in *Gata4* cKO testes was comparable to that in control testes due to the addition of CXCL12 or CCL9 (Figure 7C-b). After 4 weeks in culture, some seminiferous tubules from chemokine-treated *Gata4* cKO testes contained germ cells arrested as spermatocytes (triangle) and other tubules were devoid of germ cells (stars) (Figure 7C-c).

To determine whether GATA4 regulates the gene expression of *Cxcl12* and *Ccl9* in Sertoli cells, we isolated and cultured primary Sertoli cells and then evaluated or attenuated the endogenous GATA4 levels via adenoviral overexpression of GATA4 or transfection with a GATA4-binding site mutant (*Gata4*^Cys294Ala^) plasmid, respectively. Similar to the well-established GATA4-targeted gene *Dmrt1*, the mRNA levels of *Cxcl12* and *Ccl9* were significantly up-regulated after GATA4 adenovirus transfection, whereas these genes were significantly down-regulated by mutant plasmid transfection. The *Wt1* gene was not regulated by GATA4; its expression was not markedly altered by transfection with the *Gata4*-expressing adenovirus or the *Gata4*^Cys294Ala^ plasmid (Figure 7D). We further showed that *Cxcl12* (*Cxcl12* Luc) and *Ccl9* (*Ccl9* Luc) tluciferase activity in TM4 cells transfected with GATA4 was increased approximately threefold and fourfold, respectively, compared to control-transfected cells (Figure 7E). Collectively, in Sertoli cells, GATA4 contributes to the establishment and maintenance of the spermatogonial progenitor niche by regulating the chemokine factors CXCL12 and CCL9.

## DISCUSSION

Several lines of evidence suggest that the Sertoli cell-enriched transcription factor GATA4 is necessary during multiple stages of embryonic gonad development, including genital ridge formation [27], sex determination [18] and testicular differentiation [21]. Although a previous study indicated that GATA4 in Sertoli cells has a slight effect on spermatogenesis (a phenotype was observed after 2.5 months) [28], our results support a much earlier requirement for GATA4 in Sertoli cells in regulating the neonatal SSC niche. In this study, we generated and analyzed Sertoli cell-specific *Gata4* knockout males and found that GATA4 deficiency compromised the size of the SSC pool, leading to a complete loss of germ cells, including SSCs. Spermatogenic failure occurred from P7 to 4 weeks in *Gata4* cKO males.

In the discussion, we focus on addressing three key issues: (i) the reason for the complete loss of germ cells by as early as the initial round of spermatogenesis in *Gata4* cKO testes; (ii) the location and correction of the defect in *Gata4* cKO testes; and (iii) the exploration of possible mechanisms by which GATA4 in Sertoli cells regulates the SSC niche.

In the first postnatal week, gonocytes directly give rise to differentiating spermatogonia and SSCs in parallel [2]. The first round of mouse spermatogenesis is initiated directly from gonocytes, without passing through SSCs. However, the subsequent rounds of spermatogenesis are derived from SSCs, which are required for continuous sperm production. We showed that neither the establishment nor the maintenance of the SSC pool was appropriately regulated in *Gata4* cKO testes (Figure 3). Meanwhile, the gonocyte-derived differentiating spermatogonia differentiated into meiotic spermatocytes and then underwent apoptosis (Figure 4). Thus, these two processes led to a complete loss of germ cells by the end of the initial wave of spermatogenesis in *Gata4* cKO males (Figure 7E).

We also evaluated other potential abnormalities in Sertoli cells, such as apoptosis, proliferation, polarity and the androgen/estrogen environment. Protein levels of apoptotic markers (FasL and Bax) and proliferative markers (PCNA and Cyclin D3) were not significantly different in Sertoli cell lysates from control and *Gata4* cKO testes (Figure S3). However, there were clear signs of Sertoli cell injury and defects in polarity in *Gata4* cKO testes after 3 weeks of age, based on the presence of vacuoles and the adluminal location of Sertoli cells. To investigate whether the SSC pool maintenance failure was due to changes in the androgen and estrogen microenvironment in the seminiferous epithelium, we measured testicular testosterone and estradiol levels at puberty and found that these levels were not significantly different between control and *Gata4* cKO mice (Figure S4A). Furthermore, the expression of AR, ERα and ERβ was not obviously altered in testis lysates from control and *Gata4* cKO mice (Figure S4B). In adult *Gata4* cKO testes, interstitial cell hyperplasia was apparent. However, at this stage, *Gata4* cKO testes were much smaller than control testes, and their weight was one-fifth that of control testes, suggesting that the apparent increase in interstitial cell number in any given section may be caused by the decrease in the total volume of the adult mutant testes.

Spermatogenesis is supported by an intimate interaction between germ cells and somatic Sertoli cells, which provides the essential microenvironment (niche) for functional spermatogenesis [29]. Disruption of spermatogenesis can therefore be caused by defects that affect either germ cells or Sertoli cells and the testicular environment. To determine the location of the defect (should be impaired Sertoli cell function) and to potentially correct this defect, testis germinal cells from *Gata4* cKO mice must be exposed to a normal testicular environment. As expected, testis germinal cells from P5 *Gata4* cKO males generated functional spermatozoa after exposure to *Kit*^W/W-v^ tubules, which represent a well-developed somatic cell environment, using transplantation technology (Figure 5). This manipulation rescued the infertile phenotype of the *Gata4* cKO mice and provided new leads for the treatment of infertile patients carrying a *GATA4* mutation.

CXCL12/CXCR4 chemokine signaling, which is essential for the maintenance of SSCs [9], was attenuated in P5 *Gata4* cKO testes (Figure 6). Our microarray and RT-PCR data showed that the *Fgf2* transcript was also significantly reduced in response to GATA4 knockout in Sertoli cells (Figure 6A, 6B and Table S3). Yoon KA et al. suggested that FGF2 stimulates SDF-1 (CXCL12) expression through the *Erm* transcription factor in TM4 Sertoli cells [30]. Thus, CXCL12/CXCR4 chemokine signaling would be further abated by FGF2 attenuation in *Gata4* cKO Sertoli cells. Additional genes related to chemokine signaling that were significantly down-regulated included *Ccl9*, *Ccr1*, *Ccl3*, *Xcl1* and *Ccrl2* (Figure 6A, 6B and Table S3). However, whether these chemokines or receptors are niche signaling molecules that regulate SSC self-renewal and maintenance remains to be determined using knockout mouse models (*in vivo*) or siRNA knockdowns in SSCs (*in vitro*). In addition to the attenuated expression of niche chemokine signaling molecules, the fatty acid metabolism pathway, which is indispensable for spermatogenesis, is also dysregulated in *Gata4* cKO testes. Fatty acid enzyme genes, such as *Elovl2* [31], *Fads1*/*2* [32, 33], *Scd1*/*2* [34] and *Ppt1* [35], have been reported to be essential for spermatogenesis and Sertoli cell polarity. The transcription of *Elovl2*, *Scd1* and *Scd2* was down-regulated, whereas the *Fads1* and *Fads2* transcripts were elevated in *Gata4* cKO testes (Tables S3 and S4). The down-regulation of two genes that encode Sertoli cell-secreted factors, *Eppin* and *Nrg1*, in *Gata4* cKO testes is of particular interest, as mouse EPPIN is secreted by Sertoli cells and taken up by adjacent spermatogonia [36]. Neuregulin1 (NRG1) has only been found in Sertoli cells, and its receptor, ERBB4, localizes to the surface of spermatogonia and pre-spermatocytes [37]. Zhang et al. suggested that NRG1 promotes spermatogonia proliferation and meiotic initiation [38]. In addition, *Rhox5* [39], *Wfdc10* [40], *Mex3b* [41], *Art3* [42] and *Tubb3* [43], which are involved in spermatogenesis, were up-regulated in P5 *Gata4* cKO testes. However, the significance and mechanisms of action of these transcripts in relation to the SSC pool and spermatogenesis require further investigation. During the first wave of spermatogenesis, *Gata4* cKO testes exhibited an extensive loss of germ cells, including spermatogonia. This phenotype is further supported by the fact that transcripts related to cellular apoptosis, such as *Gpnmb* [44], *Naip5*, *Pxt1*, *Phlda1*, *Npvf*, *Igfbp3*, *Tbx3* and *Aifm2*, were up-regulated in P5 *Gata4* cKO testes.

Our study suggested that the addition of CXCL12 and CCL9 to an *in vitro* testis tissue culture system increased the number of PLZF^+^ undifferentiated spermatogonia in *Gata4* cKO testes. We performed q-PCR to determine whether the expression of the differentially expressed genes discovered in the microarray experiment could be corrected by CXCL12 and CCL9 treatment for 4 days. We randomly selected twenty Sertoli cell-expressed genes and showed that the mRNA levels of sixteen genes were significantly restored in chemokine-treated cKO testes (Figure S5). We could not exclude the possibility that other genes may be regulated by GATA4 in a chemokine-independent manner. Meanwhile, CXCL12 and CCL9 could not replace all of the functions of GATA4, but supplementation with CXCL12 and CCL9 did increase the number of PLZF^+^ undifferentiated spermatogonia. In fact, seminiferous tubules of chemokine-treated *Gata4* cKO testes either contained germ cells arrested as spermatocytes or were devoid of germ cells after 4 weeks in culture (Figure 7C).

Another group reported that GATA4 regulates Sertoli cell function and plays a role in fertility in adult male mice using the anti-Müllerian hormone receptor type 2 (*Amhr2*)-Cre system [28]. In their study, *Gata4*^flox/flox^, *Amhr2*-Cre mice developed age-dependent Sertoli cell vacuolation, exhibited increased permeability of the blood-testis barrier, and showed a loss of fertility after 2.5 months. However, in our *Gata4*^flox/flox^, *Amh*-Cre model, a complete loss of germ cells, including spermatogonia, occurred as early as 3 weeks (Figure 2). Differences between the *Amhr2*-Cre and *Amh*-Cre systems may account for these discrepant conclusions. *Amhr2*-Cre knock-in mice have been reported to exhibit Cre activity exclusively within the Müllerian duct mesenchyme at E12.5 [45, 46] and in postnatal ovarian granulosa cells [47, 48] and uterine epithelial cells [49]. Accordingly, the role of GATA4 expression in Sertoli cells in testicular development cannot be conclusively ascertained from the *Gata4*^flox/flox^, *Amhr2*-Cre model due to the non-specificity and inefficiency of *Amhr2*-Cre within Sertoli cells. In contrast, numerous studies have demonstrated that *Amh*-Cre models exhibit Cre activity in Sertoli cells after E14.5 [50-54].

In summary, we found that Sertoli cell-specific *Gata4* knockout males exhibited an extensive loss of germ cells, including spermatogonia, during the initial waves of spermatogenesis. Our results further demonstrated that GATA4 plays a critical role in the regulation of the Sertoli cell-promoted SSC niche via the transcriptional regulation of chemokine signals.

## MATERIALS AND METHODS

### Experimental animals

*Gata4*^flox/flox^ mice homozygous for a floxp allele of *Gata4*, *Amh*-Cre were used in the present study, which have been described previously [50, 53, 55]. Exons 3-5 of the *Gata4* gene was left flanked by single loxP sites after the floxed neo cassette was excised by the *in vivo* expression of Cre recombinase. *Gata4*-conditional mutants and littermate controls were obtained by crossing *Gata4*^flox/flox^ and *Gata4*^flox/+^, *Amh*-Cre mice. Genotyping was performed using PCR, as described previously [53, 55]. The primer sequences are provided in Table S1. For fertility testing, 6- to 8-week old *Gata4*^flox/flox^ (control) and *Gata4* cKO males (*n* = 10 each) were separately housed with wild-type C57BL/6 females for 3 months, and the pregnancy rate (%) was assessed. Serum and testicular testosterone (T) and estradiol (E2) concentrations were measured with a previously described tritium-based radioimmunoassay (RIA) assay [56]. All experimental protocols and animal handling procedures were conducted in accordance with the guidelines and procedures approved by the Institutional Animal Care Committee of Institute of Zoology (IOZ), University of Chinese Academy of Sciences (UCAS).

### Histological examination and immunostaining

The control and *Gata4* cKO male mice were euthanized via cervical dislocation and the testes were immediately fixed in Bouin's solution for hematoxylin and eosin (H&E) staining or in 4% formaldehyde (PFA) in PBS for immunostaining, as previously described [57-59]. In brief, tissue sections were deparaffinized and rehydrated, followed by antigen retrieval in 10 mM sodium citrate buffer. For immunohistochemistry (IHC), the sections were blocked with 5% bovine serum albumin (BSA) and incubated with the primary antibody at 4°C overnight, and then the secondary antibody was applied for 1 hour. Staining was visualized using a DAB substrate kit (Zhongshan Technology, Beijing, China). For immunofluorescence (IF), the sections were blocked using a blocking buffer (donkey serum, 0.3% Triton X-100 in PBS) and incubated with primary antibodies overnight at 4°C. Sections were washed and incubated with FITC or TRITC-conjugated secondary antibodies (Jackson ImmunoResearch, CA, USA) for 1 hour and counterstained with DAPI (Sigma, MO, USA) to identify the nuclei. The primary and secondary antibodies used for immunostaining are listed in Table S2.

### Whole-mount seminiferous tubule staining

P7 mouse testes were dissected to remove the tunica albuginea, and seminiferous tubules were untangled using forceps. Whole-mount immunofluorescence staining was performed, as described previously [60]. Stained tubules were spread on glass slides and imaged. Primary and secondary antibodies used for whole-mount seminiferous tubule IF are listed in Table S2.

### BrdU incorporation assay

BrdU labeling and detection were conducted, as previously described [27]. Briefly, mice were injected intraperitoneally (i.p.) with 100 mg/kg body weight of BrdU (Sigma, MO, USA) 4 hours before sacrifice. The testes were then removed and processed for whole-mount seminiferous tubule staining. A purified mouse anti-BrdU monoclonal antibody (BD Bioscience, CA, USA) was used for BrdU detection. To determine the percentage of GFRa1^+^ SSCs that were positive for BrdU, we counted the GFRa1^+^ SSCs in at least two 10 mm-length seminiferous tubules.

### Western blot

Western blot analysis was performed as described previously [58]. The proteins were electrophoresed under reducing conditions in 12% SDS-PAGE gels and transferred to nitrocellulose membranes. The blots were blocked in 5% BSA and incubated overnight at 4°C with the primary antibody, followed by incubation with the secondary antibody for 1 hour at room temperature. The primary and secondary antibodies used for the Western blot are listed in Table S2. The specific signals and the corresponding band intensities were evaluated using an Odyssey Infrared Imaging system and software (LI-COR Bioscience, NE, USA).

### Analysis of apoptotic cells

A TUNEL assay was conducted using an *In Situ* Cell Death Detection Kit (Promega, CA, USA), as recommended [61]. Sections were counterstained with DAPI to identify the nuclei.

### Germinal cell transplantation

Control and *Gata4* cKO male pups from the same litter were used as donor animals. Germinal cells for transplantation are obtained from the testes of mice 5 days after birth via a two-step enzymatic digestion protocol [62]. The cells were suspended in Dulbecco's modified Eagle's medium (containing 10% fetal bovine serum, 6 mM glutamine, 6 mM lactate, 0.5 mM pyruvate, 30 mg/l penicillin, 50 mg/l streptomycin and 0.03% trypan blue) at a final concentration of 10^7^ cells/ml. The cell suspension (∼10 μl) was injected into the seminiferous tubules of *Kit*^W/Wv^ recipient males (The Jackson Laboratory, ME, USA) via the rete testis, the testis were replaced, and the animals were allowed to recover. Eight weeks after transplantation, the recipient mouse testes were harvested for immunofluorescence. Spermatozoa from transplanted recipient *Kit*^W/Wv^ males were microinjected into oocytes derived from C57BL/6 female mice using a piezoelectric actuator (PrimeTech, Ibaraki, Japan). The injected oocytes were cultured and transferred to the oviducts of D1 pseudopregnant ICR female mice.

### Neonatal testis tissue culture

The testes from P3 control and *Gata4* cKO males were decapsulated and gently cut into several pieces 1-3 mm in diameter. The testis explants were cultured, as described by Sato et al. [63]. Briefly, 1.5% (w/v) agarose gel stands (10×10×5 mm in size and placed in six-well plates) were incubated with the culture medium for more than 24 hours. The testis explants were placed at the medium-air interface on each agarose gel stand and cultured at 33°C in 5% CO_2_. The medium was added to CXCL12 (GenScript, NJ, USA), CCL9 (GenScript, NJ, USA), both CXCL12 and CCL9 (final concentration = 100 ng/ml), or the vehicle control. The culture medium was replaced every 2 days. The testis explants were collected 4 days or 4 weeks later and fixed in 4% PFA.

### Sertoli cell isolation, culture and transfection

A modified previously described method was used to isolate the Sertoli cells from the testes of P5 mice [58, 64]. Briefly, the seminiferous tubules were pooled and washed with 1×PBS thrice and incubated with 1 mg/ml collagenase IV (Sigma, MO, USA), 1 mg/ml of hyaluronidase (Sigma, MO, USA) and 0.5 mg/ml DNase I (Sigma, MO, USA) in DMEM/F12 medium (HyClone, MO, USA) for 5 minutes at 37°C in a shaker. These dispersed cells were then washed twice with DMEM/F12 medium and placed into culture dishes in DMEM/F12 containing 10% fetal bovine serum (FBS) (HyClone, MO, USA) and were incubated at 37°C in 5% CO_2_. After 1 day of culture, the cells were treated with a hypotonic solution (20 mM Tris, pH 7.4) for 1 minute to remove the remaining germ cells. The GATA4 expression vector containing the Cys294Ala was generated via site-directed mutagenesis of the *Gata4*-pEGFP-N1 plasmid using a QuikChange II Site-Directed Mutagenesis Kit (Stratagene, CA, USA), according to the manufacturer's protocol. The CMV-*Gata4* adenovirus (Seven Hills Bioreagents, CA, USA) and the GATA4 binding site (Cys294Ala) mutant plasmid were used to transfect the Sertoli cells.

### Chemotaxis assays

Chemotaxis assays were performed using 8-μm-pore Cytoselect 24-well assay plates (Cell Biolabs, CA, USA), according to a previous report [11]. The Sertoli cells isolated from the P5 control and the *Gata4* cKO testes were seeded at a concentration of 2.5×10^5^ cells in the lower chambers of the transwell units and cultured in minimal media for 48 hours prior to the migration assays. Control spermatogonial progenitors (or other germ cells) from 5-day-old testes were seeded in minimal media in the upper chamber at a concentration of 5×10^5^ cells. The cells were allowed to migrate for 24 hours at 34°C, 5% CO_2_, and maximum humidity. DMEM/F12 with 10% FBS was used as a positive control in the lower chamber for all experiments, and minimal media alone, without any added cells or serum, was used as a negative control. The chemotactic index is defined as the density measurement of the migrated stem/progenitor spermatogonia compared to the negative control.

### Expression profiling

Testes were dissected from the P5 control and *Gata4* cKO mice (*n* = 3 each group) and immediately homogenized in 1 ml Tripure (Roche, Shanghai, China). Total RNA was extracted separately using an RNeasy kit (Qiagen, Suzhou, China). RNA (10 μg) was pooled, and biotinylated cRNA target was independently generated from each pool. Each cRNA was hybridized to an Affymetrix U74Av2 Murine Genome Array. A microarray analysis was performed by Beijing Yuanquanyike Biological Technology Co., LTD (Beijing, China). Screening of differentially expressed genes (up-regulation and down-regulation) were set to log2 |Fold change| ≥ 1 and a *P*-value < 0.05 and are listed in Table S3 and 4.

### Quantitative RT-PCR

RNA was extracted from the P5 testes using Trizol (Invitrogen, TX, USA) according to the manufacturer's protocol. RNA samples were subjected to reverse transcription using a PrimeScript RT reagent Kit (Takara, Dalian, China). The reactions were run in triplicate in three independent experiments. Samples CT values were normalized to the corresponding *Gapdh* CT values, and relative expression levels were calculated using the ΔΔCT method [65]. The primer sequences are provided in Table S1.

### Luciferase assay

TM4 cells were grown in F12/DMEM supplemented with 10% FBS at 37°C with 5% CO_2_. The cells were seeded at a density of approximately 50 000 cells/well in 12-well plates 12 hours before transfection. Mouse *Gata4* cDNA was amplified by PCR using testis cDNA and subcloned into the PCB6+ vector. Promoters of *Cxcl12* and *Ccl9* were amplified by PCR from mouse genomic DNA and subcloned into the pGL3-basic luciferase reporter vector (Promega, CA, USA). The cells were cotransfected with expression plasmids and the luciferase reporter plasmid. The empty expression vector (PCB6+ plasmid) was cotransfected as an internal control to allow normalization for differences in transfection efficiency [66]. The transfection was performed with Lipofectamine 2000 (Invitrogen, TX, USA) according to the manufacturer's recommendations. The cells were harvested for the luciferase assays 36 hours after transfection.

### Statistical analysis

Protein and mRNA levels, the pregnancy rate, the number of GFRa1^+^SSCs per 1-mm seminiferous tubule, the percentage of GFRa1^+^SSCs positive for BrdU, and the chemotactic index between control and *Gata4* cKO mice were analyzed using a Student's *t*-test. The results are presented as the mean±SEM. Statistical significance was considered for ^*^p < 0.05 and ^**^p < 0.01.

## SUPPLEMENTARY MATERIAL FIGURES AND TABLES


