# Supplementary material for: Loss of Gata4 in Sertoli cells impairs the spermatogonial stem cell niche and causes germ cell exhaustion by attenuating chemokine signaling
Source: Oncotarget. 2015 Oct 14;6(35):37012–27. doi: 10.18632/oncotarget.6115 (PMC4741912; doi:10.18632/oncotarget.6115)
Supplement: Supplementary file 1 [file oncotarget-06-37012-s001.pdf]

Loss of *Gata4* in Sertoli cells impairs the spermatogonial stem cell niche and causes germ cell exhaustion by attenuating chemokine signaling

Supplementary Material

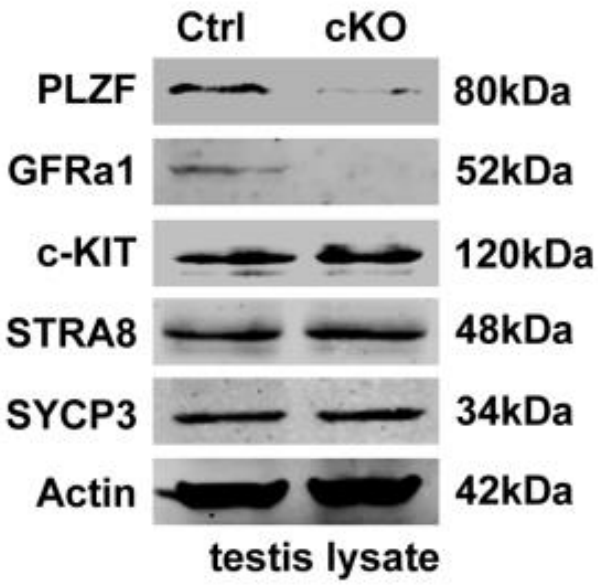

Figure S1: Western blot analysis of undifferentiated spermatogonia markers (PLZF and GFRa1), the differentiated spermatogonia marker c-KIT, and meiosis markers (STRA8 and SYCP3) in testis lysate from control and *Gata4* cKO mice. Actin served as a protein loading control.

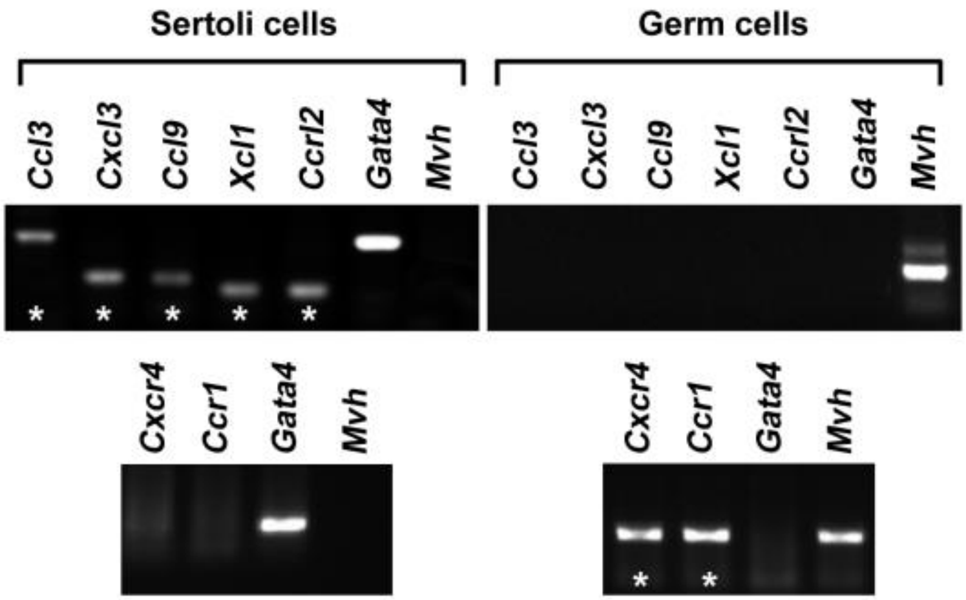

Figure S2: RT-PCR amplification of *Cxcl12*, *Ccl3*, *Ccl9*, *Xcl1*, *Ccrl2*, *Cxcr4* and *Ccr1* in cDNA samples from Sertoli cells and germ cells from P5 mice. *Gata4* is a Sertoli cell marker, and *Mvh* is a germ cell marker. A representative image from three independent experiments is shown.

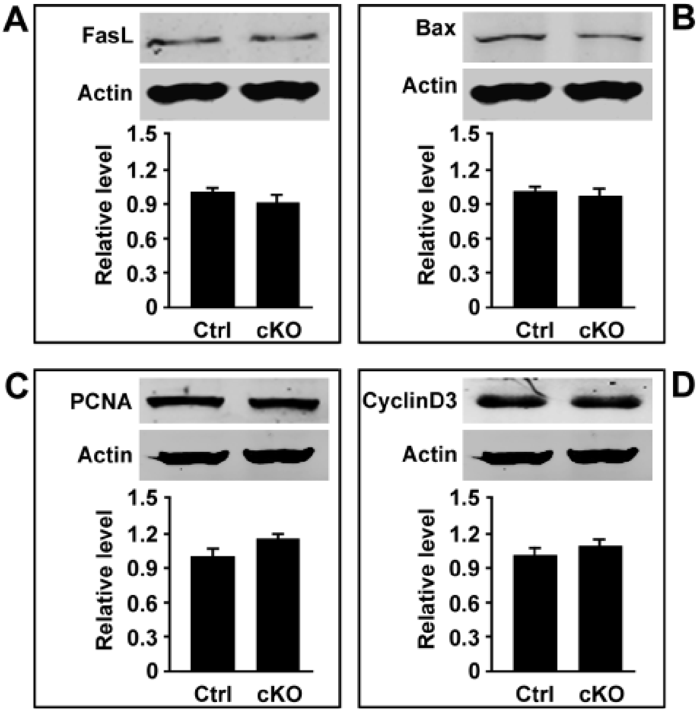

Figure S3: Western blot analysis of apoptotic markers (FasL and Bax) and proliferative markers (PCNA and Cyclin D3) in Sertoli cell lysates from control and *Gata4* cKO testes. Actin served as a protein loading control.

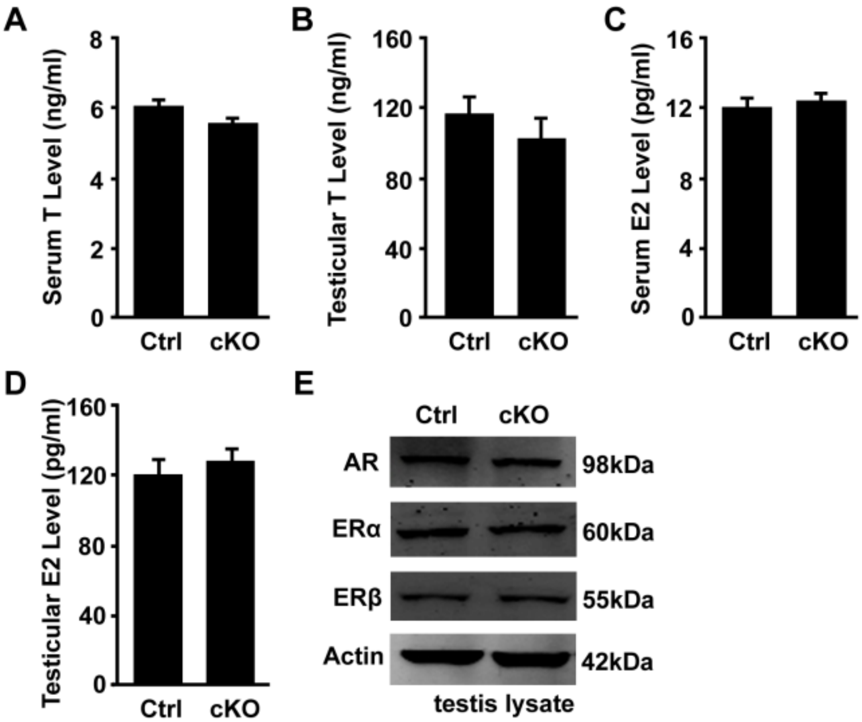

Figure S4: Serum and testicular testosterone (T) levels and estradiol (E2) levels in pubertal control and *Gata4* cKO mice (*n*=3 each group). Western blot analysis of AR, ERα and ERβ in testis lysates from control and *Gata4* cKO mice. Actin served as a protein loading control.

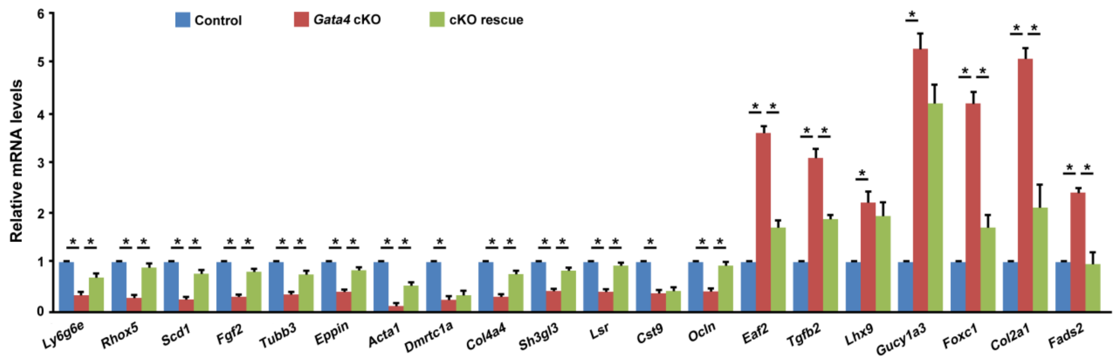

Figure S5: q-PCR analysis of twenty genes in cDNA samples from control, *Gata4* cKO and rescue testes (after CXCL12 and CCL9 treatment for 4 days). *Gapdh* served as the internal control gene. The data are expressed as the mean±S.D. *p<0.05, **p<0.01.

Supplemental Table 1: Primers used in this study.

Name		Primer sequence	Size
**Cre**	Forward:	TCCAATTTACTGACCGTACACCAA	550bp
	Reverse:	CCTGTACCTGGCAATTTCGGCTA	
**flox**	Forward:	CCC AGT AAA GAA GTC AGC ACA AGG AAA C	flox/flox= ~455 bp<br>+/flox = 355 bp and ~455 bp<br>+/+ = 355 bp
	Reverse:	AGA CTA TTG ATC CCG GAG TGA ACA TT	
Wt1	Forward:	AATGACCTCCCAGCTTGAATG	192bp
	Reverse:	CCGTGGGTGTGTATTCTGTACT	
Mvh	Forward:	TGTGGTCATATATGGAGGAAC	172bp
	Reverse:	ATCCAACATTCGATCAGCTTC	
Fgf2	Forward:	GCGACCCACACGTCAAACTA	108bp
	Reverse:	CCGTCCATCTTCCTTCATAGC	
Cxcl12	Forward:	TGCATCAGTGACGGTAAACCA	146bp
	Reverse:	TTCTTCAGCCGTGCAACAATC	
Ccl3	Forward:	TGTACCATGACACTCTGCAAC	109bp
	Reverse:	CAACGATGAATTGGCGTGGAA	
Cxcr4	Forward:	GAGGCCAAGGAAACTGCTG	145bp
	Reverse:	GCGGTCACAGATGTACCTGTC	
Ccr1	Forward:	CTCATGCAGCATAGGAGGCTT	142bp
	Reverse:	ACATGGCATCACCAAAAATCCA	
Xcl1	Forward:	TTTGTCACCAAACGAGGACTAAA	164bp
	Reverse:	CCAGTCAGGGTTATCGCTGTG	
Ccrl2	Forward:	CCCCGGACGATGAATATGATG	170bp
	Reverse:	CACCAAGATAAACACCGCCAG	
Gata4	Forward:	CCCTACCCAGCCTACATGG	139bp
	Reverse:	ACATATCGAGATTGGGGTGTCT	
Dmrt1	Forward:	GACCCCGCCTACTACAGCA	191bp
	Reverse:	GTCTGAGCAGGCACGTAAGG	
Rhox5	Forward:	ACTCGGAAGAACAGCATGATG	204bp
	Reverse:	CCCTGGTGCCACTATCCTT	
Scd1	Forward:	TTCTTGCGATACACTCTGGTGC	104bp
	Reverse:	CGGGATTGAATGTTCTTGTCGT	
Tubb3	Forward:	TAGACCCCAGCGGCAACTAT	127bp
	Reverse:	GTTCCAGGTTCCAAGTCCACC	
Eppin	Forward:	CCAGGGACCTAGTCTAGCTGA	219bp
	Reverse:	CGACGAAAGTAAGCCATACAGT	
Acta1	Forward:	CCCAAAGCTAACCGGGAGAAG	134bp
	Reverse:	CCAGAATCCAACACGATGCC	
Dmrtc1a	Forward:	GGAACCTCGTAAGGACTTTTCTC	123bp
	Reverse:	TGCATGTGTGATGGATGAGCA	
Col4a4	Forward:	GCCTGGTGTCGGGATCAAAG	211bp
	Reverse:	AGCTGGAGTCAACAAAATGCC	
Tgfb2	Forward:	TCGACATGGATCAGTTTATGCG	147bp
	Reverse:	CCCTGGTACTGTTGTAGATGGA	
Foxc1	Forward:	CCCCGGACAAGAAGATCACTC	109bp
	Reverse:	AGGTTGTGCCGTATGCTGTTC	
Eaf2	Forward:	CCAGCGGGACTTGCATACC	153bp
	Reverse:	GCCAACCTCAAGATTTCCTTCAC	
Fads2	Forward:	AAGGGAGGTAACCAGGGAGAG	150bp
	Reverse:	CCGCTGGGACCATTTGGTAA	
Gapdh	Forward:	TGGATTTGGACGCATTGGTC	211bp
	Reverse:	TTTGCACTGGTACGTGTTGAT	

Supplemental Table 2: Primary and secondary antibodies used in this study.

Primary Antibody	Source	Code	Application
Goat anti-GATA4	Santa Cruz	sc-1237	IHC/WB
Rabbit anti-WT1	Epitomics	2797-1	IHC
Rabbit anti-MVH	Abcam	ab13840	IHC
Goat anti-GFRa1	R&D Systems	AF560	IF/WB
Mouse anti-PLZF	Santa Cruz	sc-28319	IF/WB
Rabbit anti-STRA8	Abcam	ab49602	IF/WB
Rabbit anti-c-KIT	Cell Signaling	#3074	IF/WB
Mouse anti-γ-H2AX	Abcam	ab26350	IF
Rabbit anti-SYCP3	Abcam	ab15093	IF/WB
Goat anti-CXCL12	Santa Cruz	sc-6193	IF
Rabbit anti-CXCR4	Abcam	ab124824	IF
Rabbit anti-CCL9	R&D Systems	AF463	IF
Mouse anti-CCR1	R&D Systems	MAB5986	IF
Rabbit anti-TRS4	Our lab		IF
Rabbit anti-AR	Abcam	ab133273	WB
Mouse anti-ERα	Abcam	ab16460	WB
Rabbit anti-ERβ	Abcam	ab3577	WB
Rabbit anti-FasL	Abcam	ab68338	WB
Rabbit anti-Bax	Abcam	ab7977	WB
Rabbit anti-PCNA	Abcam	ab18197	WB
Rabbit anti-Cyclin D3	Abcam	ab52598	WB

Secondary Antibody	Source	Code	Application
Anti Rabbit IgG/biotin	Zhong Shan	ZB-2010	IHC
Anti Goat IgG/biotin	Zhong Shan	ZB-2050	IHC
Anti Goat IgG/TRITC	Invitrogen	A11058	IF
Anti Mouse IgG/FITC	Cell Signaling	#4408	IF
Anti Mouse IgG/TRITC	Cell Signaling	#8890	IF
Anti Rabbit IgG/FITC	Cell Signaling	#4412	IF
Anti Rabbit IgG/TRITC	Cell Signaling	#8889	IF
Anti Goat Dye 800CW	LI-COR	926-32214	WB

Supplemental Table 3: Down-regulated genes in *Gata4* cKO compared to control testes at P5.

Gene	Description	NCBI gene ID	Function	Log fold change WT to cKO	P-value WT to cKO
Spink8	Serine peptidase inhibitor, Kazal type 8	78709	Protease inhibitors	27.9	1.65×10^{-5}
Pcp4	Purkinje cell protein 4	18546	Cell migration/chemotaxis	14.0	1.38×10^{-5}
Cxcr4	Chemokine (C-X-C motif) receptor 4	12767	Chemokine receptor	13.5	4.30×10^{-4}
Acta1	Actin, alpha 1, skeletal muscle	11459	Cytoskeletal organization	8.0	6.69×10^{-3}
Enpp2	Ectonucleotide pyrophosphatase/phosphodiesterase 2	18606	Lipid signaling molecule	5.0	5.10×10^{-4}
Mybpc1	Myosin binding protein C, slow-type	109272	Cell movement	4.6	2.24×10^{-3}
Ccl3	Chemokine (C-C motif) ligand 3	20302	Chemokines	3.6	1.79×10^{-3}
Rnf138rt1	Ring finger protein 138 pseudogene	74264	Ring finger protein	4.5	3.35×10^{-4}
Hoxa10	Homeobox A10	15395	Testicular descent	4.3	3.36×10^{-3}
Dmrtc1a	DMRT-like family C1a	70887	Sertoli transcription factor	4.3	5.47×10^{-4}
Clec12b	C-type lectin domain family 12, member B	71183	Cell surface receptor	4.1	7.01×10^{-4}
Wfdc10	WAP four-disulfide core domain 10	629756	Protease inhibitors	4.1	6.36×10^{-3}
Col4a4	Collagen, type IV, alpha 4	12839	Extracellular matrix	4.0	1.59×10^{-3}
Ccl9	Chemokine (C-C motif) ligand 9	20308	Chemokines	4.0	2.10×10^{-3}
Bmp2k	BMP2 inducible kinase	140780	Protein kinase	3.8	1.32×10^{-3}
Kctd8	K^+ channel tetramerisation domain containing 8	243043	Subunit of GABA-B receptor	3.8	7.64×10^{-3}
Akr1c18	Aldo-keto reductase family 1, member C18	105349	Spermatogonia metabolism	3.7	2.51×10^{-3}
Cxcl12	Chemokine (C-X-C motif) ligand 12	20315	Chemokines	4.6	6.80×10^{-3}
Map7	Microtubule-associated protein 7	17761	Stabilizing microtubules	3.5	8.04×10^{-4}
Scd1	Stearoyl-Coenzyme A desaturase 1	20249	Fatty acid metabolism	3.5	1.84×10^{-3}
Rhox5	Reproductive homeobox 5	18617	Sertoli cell metabolism	3.5	2.49×10^{-3}
Nags	N-acetylglutamate synthase	217214	Mitochondrial matrix	3.5	2.19×10^{-3}
Ly6g6e	Lymphocyte antigen 6 complex, locus G6E	70274	Signal transduction	3.4	1.92×10^{-3}
Fgf2	Fibroblast growth factor 2	14173	SSC self-renew	3.4	1.43×10^{-3}
Sh3gl3	SH3-domain GRB2-like 3	20408	Cytoskeletal protein	3.4	1.37×10^{-3}
Zic3	Zinc finger protein of the cerebellum 3	22773	Pluripotency maintenance	3.2	1.71×10^{-3}
Ccr1	Chemokine (C-C motif) receptor 1	12768	Chemokine receptor	3.2	7.67×10^{-3}
Xcl1	Chemokine (C motif) ligand 1	16963	Chemokines	3.1	1.56×10^{-3}
Qpct	Glutaminyl-peptide cyclotransferase	70536	Glutaminyl cyclase	3.0	1.85×10^{-3}
Gata1	GATA binding protein 1	14460	Sertoli transcriptional factor	3.0	4.19×10^{-3}
Elovl2	ELOVL fatty acid elongase 2	54326	Fatty acid elongation	3.0	3.59×10^{-3}
Tcl1	T cell lymphoma breakpoint 1	21432	Akt1 associate	2.9	4.98×10^{-3}
Wnk4	WNK lysine deficient protein kinase 3	69847	Transcellular Ca^{2+} transport	2.9	3.88×10^{-3}
Ccrl2	Chemokine (C-C motif) receptor-like 2	54199	Chemokine receptor	2.9	2.28×10^{-3}
Rarres1	Retinoic acid receptor responder 1	109222	RA receptor-responsive gene	2.8	4.08×10^{-3}
Rab20	RAB20, member RAS oncogene family	19332	GTP-binding	2.8	3.30×10^{-3}
Lsr	Lipolysis stimulated lipoprotein receptor	54135	Tight junction formation	2.8	5.34×10^{-3}
Cst9	Cystatin 9	13013	Sertoli transcription factor	2.7	2.90×10^{-3}
Pip5k1b	Phosphatidylinositol-4-phosphate 5-kinase, type 1b	18719	Signaling phospholipid	2.7	4.67×10^{-4}
Adh4	Alcohol dehydrogenase 4 (class II), pi polypeptide	26876	Retinoic acid (RA) production	2.7	1.83×10^{-3}
Brwd3	Bromodomain and WD repeat domain containing 3	382236	Chromatin-modification	2.7	1.92×10^{-3}
Rragb	Ras-related GTP binding B	245670	mTOR activation	2.7	5.47×10^{-3}
Ap1m2	Adaptor protein complex AP-1, mu 2 subunit	11768	CYP26A1-responsive gene	2.6	1.35×10^{-3}
Flrt1	Fibronectin leucine rich transmembrane protein 1	396184	AR- and RB-responsive gene	2.6	7.31×10^{-3}
Etd	Embryonic testis differentiation	69501	Sertoli transcriptional factor	2.6	4.82×10^{-3}
Chp2	Calcineurin-like EF hand protein 2	70261	Cell pH regulation	2.6	6.73×10^{-3}
Nxf3	Nuclear RNA export factor 3	245610	Sertoli cell maturation	2.6	1.69×10^{-3}
Fzd5	Frizzled homolog 5 (Drosophila)	14367	Wnt5a receptor in SSC	2.6	1.02×10^{-3}
Ppt1	Palmitoyl-protein thioesterase 1	19063	Sertoli lipoprotein metabolism	2.6	5.60×10^{-3}
Tubb3	Tubulin, beta 3 class III	22152	Sertoli cell cytoskeleton	2.5	8.07×10^{-3}
Mex3b	Mex3 homolog B (C. elegans)	108797	Sertoli cell adhesion	2.5	1.38×10^{-3}
Dmrt1	Doublesex and mab-3 related transcription factor 1	50796	Sertoli transcriptional factor	2.5	8.30×10^{-3}
Scd2	Stearoyl-Coenzyme A desaturase 2	20250	Fatty acid metabolism	2.4	1.25×10^{-3}
Irs2	Insulin receptor substrate 2	384783	IGF signalling	2.4	2.50×10^{-3}
Ocln	Occludin	18260	Tight junction	2.4	3.67×10^{-3}
Snx7	Sorting nexin 7	76561	Trafficking	2.4	1.26×10^{-3}
Nrg1	Neuregulin 1	211323	Spermatoginia proliferation	2.4	8.81×10^{-3}
Art3	ADP-ribosyltransferase 3	109979	Protein modification	2.3	1.29×10^{-3}
Eppin	Epididymal peptidase inhibitor	75526	Protease inhibitors	2.3	1.28×10^{-3}
Gstm6	Glutathione S-transferase, mu 6	14867	Sertoli transcriptional factor	2.2	1.23×10^{-3}
Tex12	Testis expressed gene 12	66654	Synapsed axes	2.2	1.90×10^{-3}
Crim1	Cysteine rich transmembrane BMP regulator 1	50766	Sertoli transcriptional factor	2.2	3.93×10^{-3}
Vdr	Vitamin D receptor	22337	Vitamin D metabolism	2.1	2.87×10^{-3}
Col4a3	Collagen, type IV, alpha 3	12828	Extracellular matrix	2.1	2.68×10^{-3}
Rhox8	Reproductive homeobox 8	434768	Sertoli transcriptional factor	2.1	2.20×10^{-3}
Cadm1	Cell adhesion molecule 1	54725	Cell adhesion	2.1	2.45×10^{-3}
Syap1	Synapse associated protein 1	67043	Synapse	2.0	2.37×10^{-3}
Fshr	Follicle stimulating hormone receptor	14309	FSH signaling	2.0	3.54×10^{-3}

Supplemental Table 4: Up-regulated gene transcripts in P5 *Gata4* cKO compared to control testes.

Gene	Description	NCBI gene ID	Function	cKO to WT Log fold change	cKO to WT P-value
Gpnmb	Glycoprotein (transmembrane) nmb	78709	Macrophage activation & adhesion	18.3	8.19×10^{-4}
Mia1	Melanoma inhibitory activity 1	12587	Extracellular matrix	16.1	5.92×10^{-6}
Lrrtm4	Leucine rich repeat transmembrane neuronal 4	243499	Membrane protein	14.5	7.73×10^{-3}
Ucma	Upper zone of cartilage matrix associated	68527	Matrix-associated protein	13.9	6.05×10^{-4}
Tmem26	Transmembrane protein 26	327766	Transmembrane protein	13.3	8.08×10^{-4}
Col2a1	Collagen, type II, alpha 1	12824	Extracellular matrix	8.1	1.00×10^{-4}
Bhlhe22	Basic helix-loop-helix family, member e22	59058	Transcriptional factor	8.1	6.74×10^{-4}
Pcsk6	Proprotein convertase subtilisin/kexin type 6	18553	Follicular proprotein convertase	7.7	1.88×10^{-5}
Gucy1a3	Guanylate cyclase 1, soluble, alpha 3	60596	Signal transduction	7.6	1.74×10^{-5}
Mapt	Microtubule-associated protein tau	17762	Stablilizing microtubules	7.5	2.15×10^{-4}
Gabrb2	(GABA) A receptor, subunit beta 2	14401	Ligand-gated ion channel	7.4	7.06×10^{-4}
Svs6	Seminal vesicle secretory protein 6	20945	Secretion protein	7.4	2.52×10^{-3}
kcnk3	Potassium channel, subfamily K, member 3	16527	K^+ channel	7.2	5.78×10^{-5}
Naip5	NLR family, apoptosis inhibitory protein 5	17951	Apoptosis	6.9	6.72×10^{-3}
Crispld2	Cysteine-rich secretory protein LCCL domain 2	78892	Cell polarity complex	6.8	4.34×10^{-5}
Eaf2	ELL associated factor 2	106389	Androgen-regulated tumor suppressor	6.6	1.32×10^{-3}
Kcnj10	Potassium channel, subfamily J, member 10	16513	K^+ channel	6.5	7.28×10^{-3}
Ntsr1	Neurotensin receptor 1	18216	Tumorigenesis	6.3	1.80×10^{-4}
Oxtr	Oxytocin receptor	18430	Lipid metabolism	6.2	2.00×10^{-4}
Xdh	Xanthine dehydrogenase	22436	Oxidative metabolism	6.1	2.13×10^{-4}
Cdhr5	Cadherin-related family member 5	72040	Cellular junctions	6.1	4.18×10^{-4}
Svs5	Seminal vesicle secretory protein 5	20944	Unknown function	6.0	8.06×10^{-5}
Col27a1	Collagen, type XXVII, alpha 1	373864	Extracellular matrix	5.9	4.87×10^{-3}
Adh1	Alcohol dehydrogenase 1 (class I)	56734	Vitamin A metabolism	5.8	4.81×10^{-5}
Hsd3b1	Hydroxy-delta-steroid dehydrogenase, 3b-1	15492	Steroidogenesis	5.5	5.85×10^{-5}
Star	Steroidogenic acute regulatory protein	20845	Steroidogenesis	5.5	7.64×10^{-4}
Gria4	Glutamate receptor, ionotropic, AMPA4	14802	Gated ion channels	5.5	1.74×10^{-3}
Myoc	Myocilin	70536	Glucocorticoid response	5.2	2.61×10^{-3}
Tmem90b	Transmembrane protein 90B	433485	Transmembrane protein	5.0	3.41×10^{-3}
Col4a6	Collagen, type IV, alpha 6	94216	Extracellular matrix	5.0	2.08×10^{-4}
Bhmt	Betaine-homocysteine methyltransferase	12116	Folate metabolism	5.0	6.45×10^{-4}
Lgf1	Insulin-like growth factor 1	16000	Insulin/IGF signaling	4.8	7.00×10^{-4}
Uhrf2	Ubiquitin-like, containing PHD and RING finger 2	109113	E3 ubiquitin-protein ligase	4.7	1.20×10^{-3}
Tgfb2	Transforming growth factor, beta 2	21808	TGFβ/SMAD pathway, RA	4.3	3.89×10^{-4}
Pxt1	Peroxisomal, testis specific 1	69307	Germ cell apoptosis	4.3	3.20×10^{-4}
Foxc1	Forkhead box C1	17300	Granulosa transcription factor	4.2	2.51×10^{-3}
Cst8	Cystatin 8	13012	Degradation	4.2	3.74×10^{-4}
Apoc1	Apolipoprotein C-I	11812	Lipid metabolism	3.9	1.07×10^{-3}
Car12	Carbonic anyhydrase 12	76459	Zinc metalloenzyme	3.7	7.19×10^{-4}
Vldlr	Very low density lipoprotein receptor	22359	Lipoprotein metabolism	3.7	8.69×10^{-4}
Sftpc	Surfactant associated protein C	20389	Retinoic Acid-regulated gene	3.4	2.79×10^{-3}
Phlda1	Pleckstrin homology-like domain, A,1	21664	Apoptosis	3.3	7.67×10^{-3}
Rai2	Retinoic acid induced 2	24004	Retinoic Acid-regulated gene	3.2	1.06×10^{-3}
Npvf	Neuropeptide VF precursor	60531	Apoptosis	3.2	2.24×10^{-3}
Amer2	APC membrane recruitment 2	72125	Wnt/β-catenin signaling	3.1	1.52×10^{-3}
Hmgcs2	Hydroxy-methylglutaryl-Coenzyme A synthase 2	15360	Cholesterol biosynthesis	3.1	1.65×10^{-3}
Tspan15	Tetraspanin 15	70423	Extracellular matrix	3.0	1.83×10^{-3}
Lhx9	LIM homeobox protein 9	16876	Sertoli transcrpition factor	2.8	5.37×10^{-3}
Pik3r1	PI3K, regulatory subunit, polypeptide 1	18708	PI3K signaling	2.7	5.47×10^{-3}
Pde4d	Phosphodiesterase 4D, cAMP specific	238871	cAMP-catabolizing enzyme	2.6	3.00×10^{-3}
Igfbp3	Insulin-like growth factor binding protein 3	16009	Apoptosis	2.5	6.34×10^{-3}
Cpxm1	Carboxypeptidase X 1 (M14 family)	56264	Rhox5-negative regulated gene	2.5	6.72×10^{-3}
Col4a6	Collagen, type IV, alpha 6	94216	Extracellular matrix	2.4	1.92×10^{-3}
Tbx3	T-box 3	21386	Apoptosis	2.3	2.41×10^{-3}
Trim9	Tripartite motif-containing 9	94090	E3 ubiquitin-protein ligase	2.3	1.91×10^{-3}
Col9a2	Collagen, type IX, alpha 2	12840	Extracellular matrix	2.3	2.13×10^{-3}
Ctsd	Cathepsin D	13033	Lysosomal apoptosis	2.3	1.08×10^{-3}
Lrp1	Low density lipoprotein receptor protein 1	16971	Sertoli endocytosis	2.2	1.28×10^{-3}
Fads2	Fatty acid desaturase 2	56473	Fatty acid synthesis	2.2	1.64×10^{-3}
Prom1	Prominin 1	19126	Testicular seminoma	2.2	2.71×10^{-3}
Smoc1	SPARC related modular calcium binding 1	64075	Sertoli matricellular protein	2.2	1.59×10^{-3}
Fads1	Fatty acid desaturase 1	76267	Fatty acid synthesis	2.1	1.81×10^{-3}
Ppp1r9b	Protein phosphatase 1, regulatory subunit 9B	217124	Retinoic Acid-regulated gene	2.1	2.78×10^{-3}
Aifm2	Apoptosis-inducing factor, mitochondrion 2	71361	Apoptosis	2.0	3.78×10^{-3}
